# Length at birth z-score is inversely associated with an increased risk of bronchopulmonary dysplasia or death in preterm infants born before 32 gestational weeks: A nationwide cohort study

**DOI:** 10.1371/journal.pone.0217739

**Published:** 2019-05-31

**Authors:** Young Hwa Jung, Youngmi Park, Beyong Il Kim, Chang Won Choi

**Affiliations:** 1 Department of Pediatrics, Seoul National University Bundang Hospital, Seongnam-si, Kyunggi-do, Korea; 2 Medical Research Collaboration Center, Seoul National University Bundang Hospital, Seongnam, Korea; 3 Department of Pediatrics, Seoul National University College of Medicine, Seoul, Korea; Centre Hospitalier Universitaire Vaudois, FRANCE

## Abstract

**Objective:**

We investigated whether the extent of fetal growth restriction (FGR) in terms of not only birth weight but length and head circumference at birth is correlated with an increased risk of bronchopulmonary dysplasia (BPD) in preterm infants.

**Study design:**

A total of 4,940 very low birth weight (VLBW) infants born between 23 and 31 weeks of gestation from 2013 to 2015 who were registered in the Korean Neonatal Network (KNN) database were enrolled. Infants with major congenital malformations and those with incomplete data were excluded. Z-scores for weight, length, and head circumference at birth were calculated from the Fenton 2013 growth curve. Multivariable logistic regression analysis was performed to determine whether the z-score for length at birth was associated with BPD or death before 36 postmenstrual weeks.

**Results:**

A total of 4,662 VLBW infants were analyzed: 518 infants died before 36 postmenstrual weeks; 1,388 infants developed BPD. Decreased length at birth z-scores were significantly associated with an increased risk of BPD or death when adjusted for covariates (odds ratio (OR) 1.25 per 1-point decrease of length at birth z-score, 95% confidence interval (CI) 1.14–1.37). The association was particularly evident in infants born earlier than 29 weeks of gestation (OR 1.57, 95% CI 1.31–1.89 in infants born at 23–25 weeks; OR 1.24, 95% CI 1.09–1.42 in infants born at 26–28 weeks).

**Conclusion:**

Length at birth was inversely associated with an increased risk of BPD or death in VLBW infants born earlier than 32 weeks of gestation.

## Introduction

Bronchopulmonary dysplasia (BPD) is a major complication in preterm infants. Although mortality rates among extremely premature infants have decreased dramatically, the number of infants with BPD has increased. Premature infants with BPD require a longer hospitalization than premature infants without BPD, and BPD remains a substantial lifelong burden. [1] Specific treatment is not yet available for BPD, and early detection and multi-faceted intervention are crucial. Identifying earlier predictors of BPD is crucial for initiating preventive strategies in selected high-risk infants. Numerous studies have investigated the risk factors of BPD. [2–5] Very low gestational age (GA) and very low birth weight (VLBW) have been the most consistent and strongest risk factors for BPD. Considering that BPD is basically a developmental disorder that occurs predominantly in very premature infants, it is natural that very low GA and VLBW are the most fundamental risk factors for BPD. However, some VLBW infants are born at relatively later GAs. These small-for-gestational-age (SGA) infants are most likely the result of fetal growth restriction (FGR). FGR develops for a variety of reasons and most likely reflects fetal adaptation to an unfavorable intrauterine environment. [6] Growth restriction during fetal life is known to reduce the capacity for normal growth after birth and to lead to permanent alterations in metabolism, development, and ultimate growth potential. [7–10] Recently, FGR and its consequence, SGA, have been suggested as having a particular role in the development of BPD. [11–13] The pathologic hallmark of BPD is an arrest of alveolar and pulmonary vascular development. As the growth and development of the fetal lung might be adversely affected by FGR due to its global impact on fetal growth and development, a link between FGR and BPD can be speculated.

In a previous study, SGA infants (birth weight <10^th^ percentile) had a significantly greater risk of developing BPD compared with the reference group (birth weight between the 25^th^ and 75^th^ percentiles). [8] Carl Bose et al reported that birth weight z-scores provided the best information on the risk of BPD among prenatal factors in preterm infants born earlier than 28 week of gestation. [12] Most reports demonstrating the association of FGR with BPD have primarily focused on birth weight. Few studies have evaluated the associations of lengthwise growth restriction with BPD.

In the present study, we investigated whether the extent of FGR in terms of not only birth weight but also length and head circumference at birth was associated with increased risk of BPD or death before 36 weeks postmenstrual age (PMA) using a nationwide cohort of VLBW infants. We also assessed the relative impacts of the z-scores of these anthropometric measurements at birth on BPD or death before 36 weeks PMA in three different GA subgroups.

## Subject and methods

This was a prospective cohort study of VLBW infants (<1,500 g) born between 23^+0^ (week^+day^) and 31^+6^ gestational weeks from January 2013 to December 2015 and admitted to neonatal intensive care units participating in the Korean Neonatal Network (KNN). VLBW infants with major congenital malformations were excluded. The KNN is a nationwide VLBW infant registry that collects demographic and clinical data from 69 hospitals across South Korea using a standardized operating procedure. [14] Approximately 70% of the VLBW infants born in South Korea are registered in the KNN. The KNN registry was approved by the institutional review board, and written informed consent was obtained from the parents upon enrollment at each participating hospital.

The KNN registry collects prenatal and neonatal data; the collected prenatal data include pregnancy-induced hypertension (PIH), oligohydramnios, clinical and histological chorioamnionitis, and administration of prenatal corticosteroids. The collected neonatal data include GA; weight, length and head circumference at birth; gender; and clinical information on respiratory distress syndrome (RDS), patent ductus arteriosus (PDA), culture-proven sepsis, and total duration of mechanical ventilation. The z-scores for weight, length, and head circumference at birth were calculated from the Fenton 2013 growth curve. Regarding the administration of prenatal corticosteroids, only cases in which prenatal corticosteroid therapy (betamethasone or dexamethasone) was completed within 7 days before delivery were counted. RDS was defined as a respiratory insufficiency that manifested at or shortly after birth, was accompanied by typical radiologic findings compatible with RDS, and required surfactant replacement therapy.

The primary outcome was BPD or death before 36 weeks PMA. BPD was defined as the need for supplemental oxygen at 36 weeks PMA using the National Institute of Child Health and Human Development criteria. [15]

Because GA has a substantial influence on the primary outcome, the study population was divided into three GA groups (23^+0^–25^+6^ weeks, 26^+0^–28^+6^ weeks and 29^+0^–31^+6^ weeks). The associations of anthropometric measurement values at birth and prenatal factors with BPD or death before 36 weeks PMA were evaluated in univariate analyses. Covariates were included in the multivariable logistic regression models if they were associated with the outcome at *P*<0.2 in univariate analyses. To reduce multicollinearity, which may be present between the anthropometric data at birth and their z-scores, we included only the z-scores of the anthropometric measurement values. To investigate whether the z-scores for weight, length and head circumference at birth are independently associated with BPD or death before 36 weeks PMA, multivariable logistic regression analyses with backward elimination were performed.

For comparisons of categorical variables, chi-squared and Fisher’s exact tests were performed. Continuous variables were compared using independent *t*-tests, the Mann-Whitney test, and Kruskal-Wallis test. *P*<0.05 was considered statistically significant. The results are expressed as odds ratios (ORs) and 95% confidence intervals (CIs). All statistical analyses were performed using SPSS ver. 22.0 software (IBM SPSS Statistics, Armonk, NY)

## Results

**Fig 1:**
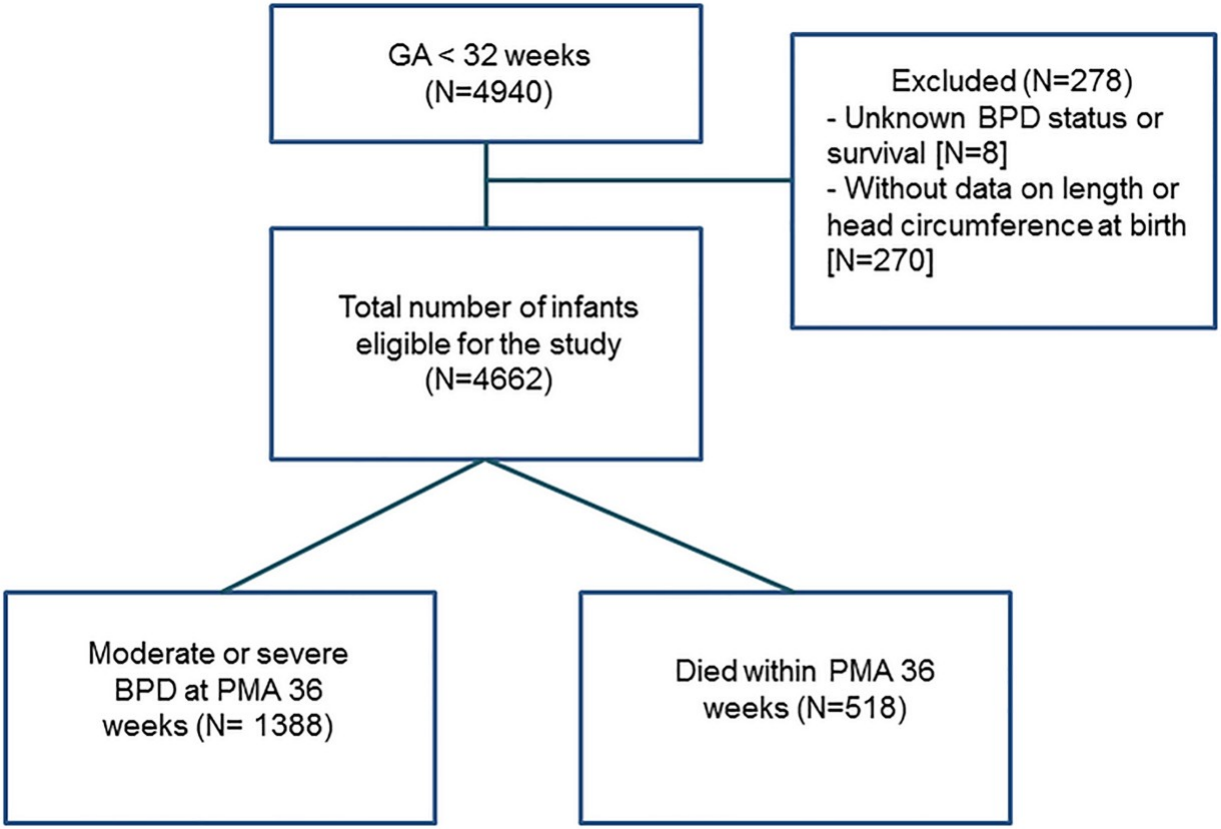
Flow diagram for the study population.

A flow diagram of our study is presented in Fig 1. Of the preterm infants who were registered in the KNN nationwide cohort, a total of 4,940 VLBW infants who were born between 23^+0^ and 31^+6^ weeks of gestation from January 2013 to December 2015 were enrolled in this study. After excluding 8 infants with an unknown BPD status at 36 weeks PMA and 270 infants without data on length or head circumference at birth, a total of 4,662 VLBW infants were finally included in the analysis. Their mean GA was 28^+1^±2^+1^ weeks, and their mean birth weight was 1,058±271 g. Of these VLBW infants, 518 (11.1%) died before 36 weeks PMA, and 1,388 (33.5% of surviving infants) infants developed BPD.

The anthropometric measurement values at birth of the infants, and the maternal and neonatal characteristics in each GA group are displayed in Table 1. Among the 4,662 infants, 875 were born at 23^+0^–25^+6^ weeks, 1,889 at 26^+0^–28^+6^ weeks, and 1,898 at 29^+0^–31^+6^ weeks. The number of SGA infants and the proportion of infants who had any degree of FGR (z-score below -1) were highest in the later GA group (29^+0^–31^+6^ weeks).

**Table 1:** Anthropometric measurement values at birth, and the maternal and neonatal characteristics in each gestational age group.

	23^+0^–25^+6^ weeks (n = 875)	26^+0^–28^+6^ weeks (n = 1,889)	29^+0^–31^+6^ weeks (n = 1,898)	*P*-value
Birth weight (g), mean±SD	732±145	1,021±209	1,245±204	<0.001
Length at birth (cm), mean±SD	32.0±2.5	35.7±2.9	38.3±2.7	<0.001
HC (cm), mean±SD	22.5±1.6	25.1±1.7	27.2±1.6	<0.001
Male, n (%)	467 (53.4)	993 (52.6)	944 (49.7)	0.108
SGA (<10^th^ percentile), n (%)	70 (8.0)	140 (7.4)	269 (14.2)	<0.001
Birth weight z-score, mean±SD	0.189±0.960	0.114±0.869	-0.448±0.742	<0.001^‡^
*<-2*, *n (%)*	27 (3.1)	46 (2.4)	61 (3.2)	
*-2—1*, *n (%)*	66 (7.5)	154 (8.1)	362 (19.1)	
*≥-1*, *n (%)*	782 (89.4)	1,689 (89.4)	1,475 (77.7)	
Length at birth z-score, mean±SD	0.112±1.078	0.069±1.098	-0.356±1.110	<0.001^‡^
*<-2*, *n (%)*	30 (3.4)	98 (5.2)	145 (7.6)	
*-2—1*, *n (%)*	82 (9.4)	153 (8.1)	309 (16.3)	
*≥-1*, *n (%)*	763 (87.2)	1,638 (86.7)	1,444 (76.1)	
HC at birth z-score, mean±SD	0.114±1.125	0.174±1.361	-0.155±0.376	<0.001^‡^
*<-2*, *n (%)*	27 (3.1)	65 (3.4)	122 (6.4)	
*-2—1*, *n (%)*	75 (8.6)	195 (10.3)	273 (14.4)	
*≥-1*, *n (%)*	773 (88.3)	1,629 (86.2)	1,503 (79.2)	
PIH, n (%)	68 (7.8)	276 (14.6)	478 (25.2)	<0.001
Oligohydramnios, n (%)*	125 (15.9)	229 (13.3)	218 (12.4)	0.053
Prenatal corticosteroids, n (%)**	367 (42.4)	870 (46.8)	920 (49.3)	0.004
RDS, n (%)	858 (98.1)	1,796 (95.1)	1,410 (74.3)	<0.001
Moderate or severe BPD, n (%)^†^	404 (67.0)	656 (38.6)	328 (17.8)	<0.001
Death <36 weeks PMA, n (%)	272 (31.1)	188 (10.0)	58 (3.1)	<0.001
BPD or death, n (%)	677 (77.4)	844 (44.7)	386 (20.3)	<0.001

* Values are missing in 90 infants in the 23^+0^–25^+6^ weeks group, 168 infants in the 26^+0^–28^+6^ weeks group, and 138 infants in the 29^+0^–31^+6^ weeks group.

** Values are missing in 10 infants in the 23^+0^–25^+6^ weeks group, 29 infants in the 26^+0^–28^+6^ weeks group, and 138 infants in the 29^+0^–31^+6^ weeks group.

† Values are missing in 272 infants in the 23^+0^–25^+6^ weeks group, 188 infants in the 26^+0^–28^+6^ weeks group, and 58 infants in the 29^+0^–31^+6^ weeks group.

‡ *P*-value for the *Kruskal-Wallis* test.

HC, head circumference; SGA, small for gestational age; PIH, pregnancy-induced hypertension; RDS, respiratory distress syndrome; PMA, postmenstrual age; BPD, bronchopulmonary dysplasia.

**Fig 2:**
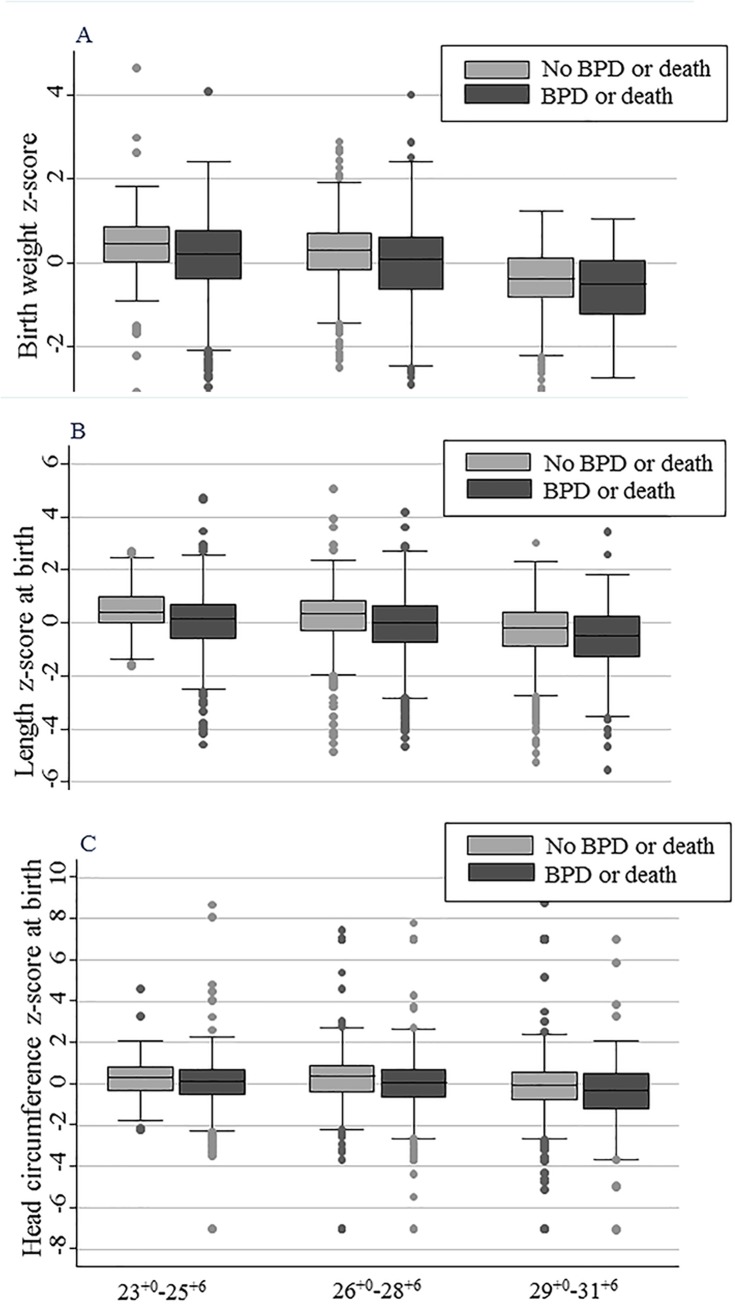
Comparisons of the z-scores for the anthropometric measurement values at birth between the infants who developed BPD or died before 36 weeks postmenstrual age and the infants who survived until 36 weeks postmenstrual age without BPD in each gestational age group. The box and whisker plots depict the comparisons of weight z-scores (A), length z-scores (B), and head circumference z-scores (C) at birth between the infants who developed BPD or died before 36 weeks postmenstrual age (BPD or death group) and the infants who survived until 36 weeks postmenstrual age without BPD (control group) in each gestational age group. The gray boxes represent the control group, and the black boxes represent the BPD or death group.

Table 2 shows the univariate analysis of the associations of the anthropometric measurement values at birth, as well as the associations of the maternal and neonatal characteristics with BPD or death before 36 weeks PMA in each GA group. Early GA, low birth weight, and low anthropometric measurement values at birth and their z-scores were significantly associated with an increased risk of BPD or death in all three GA groups. Similarly, any degree of FGR in weight, length, and head circumference (z-scores <-1) were significantly associated with an increased risk of BPD or death in all the GA groups. Among the maternal and neonatal characteristics, male gender, oligohydramnios, and RDS were significantly associated with an increased risk of BPD or death in the 26^+0^–28^+6^ weeks GA group and the 29^+0^–31^+6^ weeks GA group. PIH was significantly associated with an increased risk of BPD or death only in the 26^+0^–28^+6^ weeks GA group. The administration of prenatal corticosteroids was not associated with BPD or death in any of the GA groups. The median z-scores for weight (A), length (B), and head circumference (C) at birth were significantly lower in the infants who developed BPD or died before 36 weeks PMA than in the infants who survived until 36 weeks PMA without BPD in all of the GA groups (Fig 2).

**Table 2:** Univariate analysis of the associations of the anthropometric measurement values at birth, and the associations of the maternal and neonatal characteristics with BPD or death before 36 weeks postmenstrual age in each gestational age group.

Variables	23^+0^–25^+6^ weeks (n = 875)	26^+0^–28^+6^ weeks (n = 1,889)	29^+0^–31^+6^ weeks (n = 1,898)
	OR (95% CI)	OR (95% CI)	OR (95% CI)
Gestational age^a^	2.54 (1.97–3.29)	1.60 (1.44–1.79)	1.30 (1.14–1.50)
Birth weight^b^	1.51 (1.34–1.71)	1.29 (1.23–1.35)	1.22 (1.16–1.29)
Birth weight z-score^c^	1.44 (1.20–1.73)	1.49 (1.33–1.66)	1.45 (1.16–1.29)
Length at birth^d^	1.33 (1.23–1.44)	1.20 (1.16–1.24)	1.15 (1.10–1.19)
Length at birth z-score^c^	1.54 (1.30–1.81)	1.42 (1.30–1.55)	1.32 (1.20–1.45)
HC at birth^d^	1.33 (1.20–1.48)	1.28 (1.21–1.35)	1.25 (1.17–1.34)
HC at birth z-score^c^	1.22 (1.05–1.40)	1.20 (1.11–1.29)	1.26 (1.15–1.38)
FGR in weight (birth weight z-score <-1)	3.97 (1.81–8.73)	2.62 (1.92–3.56)	2.01 (1.56–2.57)
FGR in length (length at birth z-score <-1)	5.01 (2.29–10.95)	2.56 (1.93–3.38)	1.91 (1.49–2.43)
FGR in HC (HC at birth z-score<-1)	2.38 (1.27–4.44)	1.88 (1.44–2.45)	1.85 (1.43–2.39)
Male	1.36 (0.99–1.87)	1.33 (1.11–1.60)	1.41 (1.12–1.76)
PIH	1.26 (0.67–2.35)	1.47 (1.14–1.90)	1.10 (0.86–1.42)
Oligohydramnios*	1.34 (0.83–2.16)	1.39 (1.05–1.84)	1.52 (1.09–2.10)
Prenatal corticosteroids**	1.08 (0.79–1.50)	1.11 (0.93–1.34)	0.89 (0.71–1.11)
RDS	1.44 (0.50–4.13)	1.93 (1.23–3.04)	2.74 (2.00–3.76)

^a^Per 1 week decrease

^b^Per 100 g decrease

^c^Per 1 point decrease

^d^Per 1 cm decrease.

* Values are missing in 90 infants in the 23^+0^–25^+6^ weeks group, 168 infants in the 26^+0^–28^+6^ weeks group, and 138 infants in the 29^+0^–31^+6^ weeks group.

** Values are missing in 10 infants in the 23^+0^–25^+6^ weeks group, 29 infants in the 26^+0^–28^+6^ weeks group, and 138 infants in the 29^+0^–31^+6^ weeks group.

OR, odds ratio; CI, confidence interval; BPD, bronchopulmonary dysplasia; HC, head circumference; FGR, fetal growth restriction; PIH, pregnancy-induced hypertension; RDS, respiratory distress syndrome.

**Fig 3:**
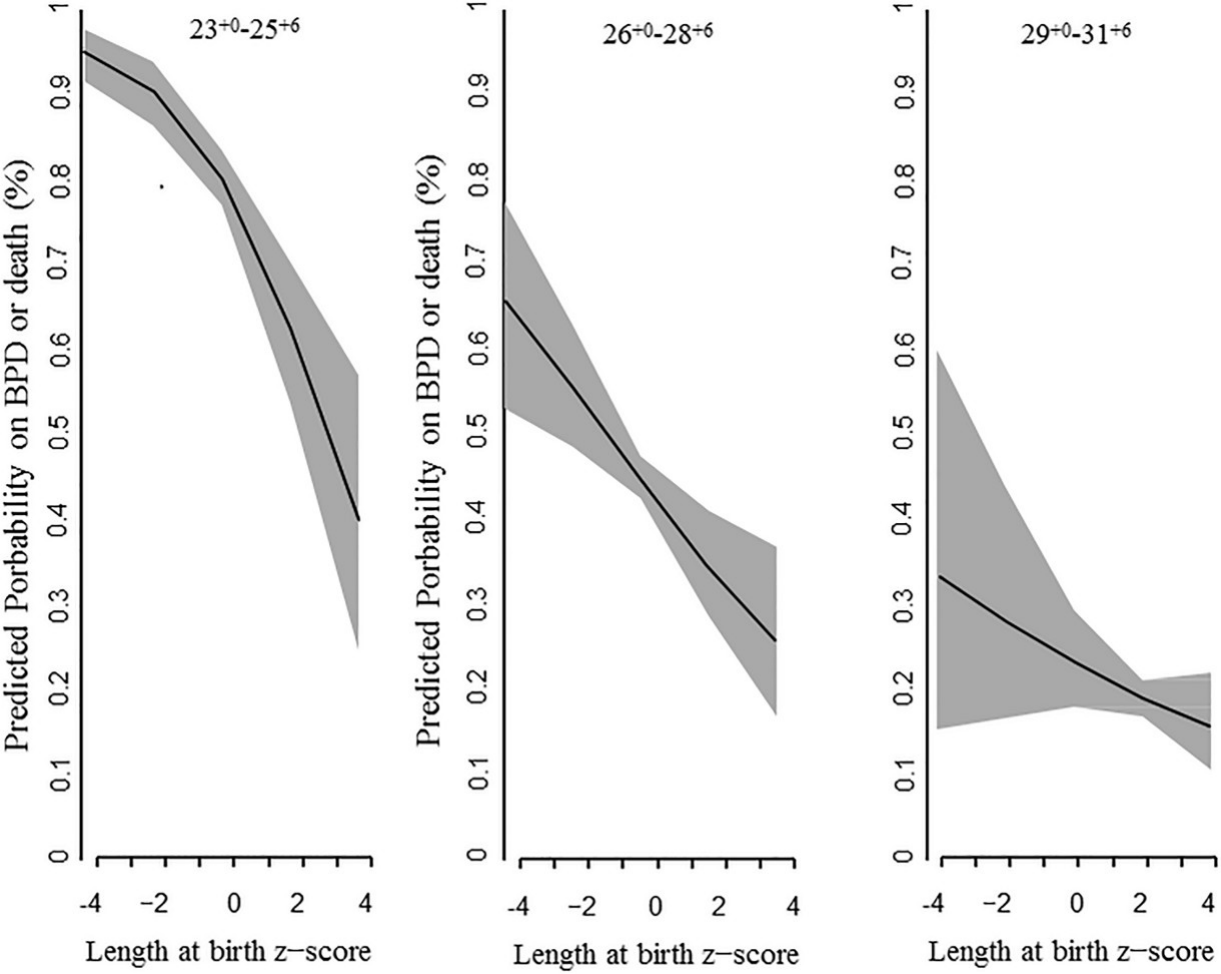
Length at birth z-score for predicting BPD or death by multivariable logistic regression analysis. The regression line and its 95% CI (gray shade) are shown for each gestational age group.

Table 3 displays the results of the multivariable logistic regression analyses for the whole population and for each GA group. The logistic regression models for the whole population and for each GA group included GA; the z-scores for birth weight, length, and head circumference at birth; male gender; PIH; oligohydramnios; and RDS. After adjusting for the above covariates, decreased length at birth z-score was significantly associated with an increased risk of BPD or death in the whole population (OR 1.25 per 1-point decrease, 95% CI 1.14–1.37), in the 23^+0^–25^+6^ weeks GA group (OR 1.57 per 1-point decrease, 95% CI 1.31–1.89), and in the 26^+0^–28^+6^ weeks GA group (OR 1.24 per 1-point decrease, 95% CI 1.09–1.42), but not in the 29^+0^–31^+6^ weeks GA group. Decreased birth weight z-score was associated with an increased risk of BPD or death in the whole population (OR 1.35 per 1-point decrease, 95% CI 1.20–1.52), in the 26^+0^–28^+6^ weeks GA group (OR 1.33 per 1-point decrease, 95% CI 1.13–1.56), and in the 29^+0^–31^+6^ weeks GA group (OR 1.38 per 1-point decrease, 95% CI 1.05–1.80), but not in the 23^+0^–25^+6^ weeks GA group. Decreased head circumference z-score was not associated with increased risk of BPD or death in any of the GA groups. In the 23^+0^–25^+6^ weeks GA group, among the z-scores for the anthropometric measurement values at birth, only the length at birth z-score was significantly associated with BPD or death. Fig 3 shows the regression line of length at birth z-score and its 95% CI for predicting the risk of BPD or death in each GA group.

**Table 3:** Multivariable logistic regression analyses of the associations of the z-scores for the anthropometric measurement values at birth, as well as the associations of the maternal and neonatal characteristics with BPD or death before 36 weeks postmenstrual age in the whole population and in each gestational age group.

Gestational age group	Variables	BPD or death odds ratio (95% confidence interval)	*P*-value
Whole population 23^+0^–31^+6^ weeks (n = 4,266)	Birth weight z-score*	1.35 (1.20–1.52)	<0.001
	Length at birth z-score*	1.25 (1.14–1.37)	<0.001
	Gestational age	1.70 (1.63–1.77)	<0.001
	Male	1.37 (1.19–1.58)	<0.001
	Oligohydramnios	1.22 (1.00–1.50)	0.056
	RDS	1.90 (1.46–2.48)	<0.001
23^+0^–25^+6^ weeks (n = 785)	Length at birth z-score*	1.57 (1.31–1.89)	<0.001
	Gestational age	2.65 (2.01–3.49)	<0.001
	Male	1.48 (1.04–2.11)	0.028
26^+0^–28^+6^ weeks (n = 1,721)	Birth weight z-score*	1.33 (1.13–1.56)	0.001
	Length at birth z-score*	1.24 (1.09–1.42)	0.001
	Gestational age	1.65 (1.47–1.85)	<0.001
	Male	1.34 (1.09–1.63)	0.004
29^+0^–31^+6^ weeks (n = 1,760)	Birth weight z-score*	1.38 (1.05–1.80)	0.020
	Length at birth z-score*	1.16 (0.99–1.36)	0.070
	Gestational age	1.44 (1.22–1.70)	<0.001
	Male	1.33 (1.04–1.69)	0.023
	RDS	2.38 (1.70–3.33)	<0.001

* Per 1-point decrease.

Other than the z-scores for the anthropometric measurement values at birth, low GA and male gender were consistently significantly associated with an increased risk of BPD or death in the whole population and in all three GA groups. RDS was significantly associated with an increased risk of BPD or death in the whole population as well as in the 29^+0^–31^+6^ weeks GA group only.

Gestational age, the z-scores for birth weight, length, and head circumference at birth, male gender, pregnancy induced hypertension, oligohydramnios, and RDS were included in the logistic regression model in the whole population and in each gestational age group. BPD, bronchopulmonary dysplasia; RDS, respiratory distress syndrome.

## Discussion

In our nationwide cohort of VLBW infants born earlier than 32 weeks of gestation, decreased birth weight z-score, decreased length at birth z-score, low GA and male gender were significantly associated with an increased risk of BPD or death before 36 weeks PMA. Interestingly, the association of decreased length at birth z-score with BPD or death was observed in VLBW infants born at an early to middle GA (≤28^+6^ weeks), whereas the association of decreased birth weight z-score with BPD or death was observed in VLBW infants born at a middle to late GA (≥26^+0^ weeks). At middle GA (26^+0^–28^+6^ weeks), both decreased length at birth z-score and decreased birth weight z-score were associated with an increased risk of BPD or death. There have been several reports that FGR or SGA is associated with an increased risk of BPD. [8–11,16,17] In general, SGA is defined as birth weight less than the 10^th^ percentile of the population-based birth weight data obtained from infants born at the same GA. Most reports have demonstrated the association of FGR with BPD by using birth weight z-scores as a parameter of FGR. However, few studies have investigated the associations of other anthropometric indices of FGR, such as length and head circumference, with BPD. In this cohort of VLBW infants born earlier than 32 weeks of gestation, we investigated whether three different types of FGR, assessed as z-scores of birth weight, length and head circumference at birth, are associated with an increased risk of BPD or death. We found that only decreased length at birth z-score, among the z-scores of the three types of anthropometric measurement values, was significantly associated with BPD or death in the early GA group, in which the risk of BPD or death was substantial; however, the birth weight z-score was not associated with BPD or death in the early GA group. This finding may be due to the fact that on average, the birth weight z-score in the early GA group was higher than that in the other two more mature GA groups. In contrast, in the late GA group in which the risk of BPD or death is quite low, only decreased birth weight z-score was significantly associated with BPD or death. Decreased head circumference at birth z-score was not associated with BPD or death in the whole population and in any of the GA groups. These findings suggest that lengthwise FGR at an earlier GA when the lungs are in the late canalicular stage may be associated with the development of BPD or death. [18]

Gender and height are the most important predictors of lung function in both children and adults. It is well known that linear relationships exist between lung function and height. [19,20] Height is also a main predictor of functional vital capacity (VC) in childhood. [21] For normal children, VC can be calculated with the equation VC = −2.41+0.0341×(Height)±2(0.199). [22] In addition, there are several reports that lung function parameters in children, including tidal volume, functional residual capacity, and airway resistance, are all significantly dependent on height. [23] However, there is a paucity of data regarding the relationship between height and lung growth and development. [24,25] The relationship between fetal length and fetal lung growth and development remains to be revealed. BPD is a developmental disorder of the lungs that occurs predominantly in premature infants. The pathologic hallmark of BPD is an arrest or lag of alveolar and pulmonary vascular growth and development. [26] The finding that decreased length at birth was associated with an increased risk of BPD or death suggests that fetal lengthwise growth might affect lung growth and development.

Among the maternal characteristics evaluated in this study, the administration of prenatal corticosteroids was not associated with BPD or death in any of the three GA groups. Oligohydramnios was not associated with BPD or death after adjusting for covariates. Several studies have demonstrated the relationship between intrauterine exposure to maternal preeclampsia and the risk of the infant developing BPD. Korhonen et al reported that preeclampsia is protective and reduces the incidence of BPD. [27] In contrast, Hansen et al and Redline et al reported that preeclampsia is associated with an increased incidence of BPD. [28,29] Recently, O’shea et al reported that preeclampsia did not significantly increase the risk of BPD in extremely preterm infants in a pooled cohort meta-analysis. [30] In our cohort, PIH was significantly associated with an increased risk of BPD or death in the univariate analysis (OR 1.47, 95% CI 1.14–1.90 in the 26^+0^–28^+6^ weeks GA group). However, the statistical association between PIH and BPD or death disappeared after adjusting for other covariates.

As mentioned in the Subject and methods section, we did not include the absolute anthropometric measurements at birth and their z-scores together in the same logistic regression models because of potential multicollinearity between them. Moreover, we aimed to assess the associations of z-scores of anthropometric measurements at birth, instead of the associations of absolute anthropometric measures at birth, with BPD or death because we focused on the extent to which FGR, reflected by z-scores, which were standardized by gender and gestational age, was associated with BPD or death.

The limitation of the present study is that the infants’ lengths at birth were not adjusted for their parents’ heights. The KNN registry does not collect parental weight or height information. Because height is strongly influenced by genetics, fetal length might be related to parental height. A database with more detailed parental information will be needed to adjust for the genetic influence of parental height on fetal length. In addition, we did not have information regarding large airway problems such as tracheobronchomalacia, which can mimic BPD, and data on the nutritional status of the infants, which might have substantially affected the postnatal growth trajectory and neonatal outcomes including BPD. Information about the hospitals where the VLBW infants were admitted was not provided to individual researchers. The raw data were provided to the individual researchers after the hospital codes were deleted. Therefore, we were not able to include the hospital as a covariate in the analyses. Moreover, we did not include many postnatal factors, including PDA, sepsis, and the duration of mechanical ventilation in the analyses. These postnatal factors can contribute to the development of BPD. [31] Information on PDA, sepsis, and the duration of mechanical ventilation is collected in the KNN registry. However, we did not include these postnatal factors in the logistic regression models in the multivariable analyses. Although PDA has often been considered a risk factor for BPD, the exact role of PDA in the development of BPD in preterm infants remains controversial. [32,33] Furthermore, there is no consensus regarding the diagnosis of hemodynamically significant PDA, and substantial controversies exist regarding when and how to treat PDA. Sepsis has also been related to the development of BPD. [34] However, it was impossible to determine whether sepsis was a contributor to BPD or a comorbidity of BPD in our cohort data because the timing of sepsis is not collected in the KNN registry. Likewise, although prolonged mechanical ventilation may predispose preterm infants to BPD [35], the total duration of mechanical ventilation might be a reflection of the BPD severity rather than a preceding factor.

In conclusion, we demonstrated that decreased fetal lengthwise growth was associated with an increased risk of BPD or death, especially in early GA infants, in a Korean nationwide cohort of VLBW infants born before 32 weeks of gestation. It can be speculated that fetal lengthwise growth is closely related with fetal lung growth and development, although the causal relationship between the two remains unrevealed. If lengthwise growth is also linked to lung growth and development during the postnatal period in preterm infants, a means of accelerating postnatal lengthwise growth could be a potential therapeutic approach for enhancing lung growth and development and thus alleviating BPD.

## Supporting information

S1 Table Maternal and neonatal characteristics between survival without BPD until 36 weeks of postmenstrual age group and BPD or death before 36 weeks of postmenstrual age group. (DOCX)
