# Supplementary material for: Length at birth z-score is inversely associated with an increased risk of bronchopulmonary dysplasia or death in preterm infants born before 32 gestational weeks: A nationwide cohort study
Source: PLoS One. 2019 May 31;14(5):e0217739. doi: 10.1371/journal.pone.0217739 (PMC6544374; doi:10.1371/journal.pone.0217739)
Supplement: S1 Table — (DOCX) [file pone.0217739.s001.docx]

**Supplementary Table 1. Maternal and neonatal characteristics between Survival without BPD until 36 weeks of postmenstrual age group and BPD or death before 36 weeks of postmenstrual age group.**

| **Variables** | **Survival without BPD**  **(n=2,755)** | **BPD or death**  **(n=1,907)** | ***P*-value** |
| --- | --- | --- | --- |
| Gestational age (weeks), mean±SD | 29.0±1.8 | 27.0±2.1 | <0.001 |
| Birth weight (g), mean±SD | 1,156±229 | 918±263 | <0.001 |
| Birth weight z-score, mean±SD | -0.099±0.805 | -0.104±0.993 | 0.859 |
| Length at birth (cm), mean±SD | 37.3±2.9 | 34.2±3.6 | <0.001 |
| Length at birth z-score, mean±SD | -0.027±1.032 | -0.195±1.230 | <0.001 |
| HC at birth (cm), mean±SD | 26.3±2.0 | 24.3±2.4 | <0.001 |
| HC at birth z-score, mean±SD | 0.101±1.328 | -0.075±1.339 | <0.001 |
| FGR in weight  (birth weight z-score <-1), n (%) | 361 (13.1) | 339 (17.8) | <0.001 |
| FGR in length  (length at birth z-score <-1), n (%) | 415 (15.1) | 395 (20.7) | <0.001 |
| FGR in HC  (HC at birth z-score <-1), n (%) | 394 (14.3) | 352 (18.5) | <0.001 |
| Male, n (%) | 1,336 (48.5) | 1,068 (56.0) | <0.001 |
| PIH, n (%) | 518 (18.8) | 304 (15.9) | 0.012 |
| Oligohydramnios, n (%)^*^ | 296 (11.6) | 276 (16.1) | <0.001 |
| Prenatal corticosteroids, n (%)^**^ | 1,298 (47.6) | 859 (46.1) | 0.299 |
| RDS, n (%) | 2,247 (81.6) | 1,817 (95.3) | <0.001 |

* Values are missing in 396 infants

** Values are missing in 177 infants

BPD, bronchopulmonary dysplasia; HC, head circumference; FGR, fetal growth restriction; PIH, pregnancy induced hypertension; RDS, respiratory distress syndrome
